# A new species of *Bembidion* ( *Ecuadion*) from Ecuador (Coleoptera, Carabidae, Bembidiini), with a key to members of the *georgeballi* species group

**DOI:** 10.3897/zookeys.249.4149

**Published:** 2012-12-10

**Authors:** David R. Maddison, Luca Toledano

**Affiliations:** 1 Department of Zoology, Oregon State University, Corvallis, OR 97331, USA; 2 Museo Civico di Storia Naturale di Verona, Lungadige Porta Vittoria 9, 37129 Verona, Italy

**Keywords:** *Bembidion*, Trechinae, Bembidiini, DNA, morphology, taxonomy, systematics

## Abstract

A new species of ground beetle, *Bembidion ricei*, is described from the Andes mountains of Ecuador east of Quito. It belongs to the *georgeballi* species group of subgenus *Ecuadion*, and is most similar to *Bembidion georgeballi*. A key to the species of the group is provided.

## Introduction

*Ecuadion* is a diverse subgenus of *Bembidion* restricted to higher elevations in South and Central America (Erwin 1982; Moret and Toledano 2002; Toledano 2008; Vigna Taglianti and Toledano 2008). Adult beetles range in length from 2.2 to 6.1 mm; they are generally shades of brown, either uniform or with various patterns, a few species having metallic reflections. Unlike many other *Bembidion*, most *Ecuadion* are not closely associated with shores of bodies of water; they inhabit the leaf litter of cloud forests (e.g., *Bembidion georgeballi* Toledano, *Bembidion onorei* Moret and Toledano, *Bembidion andersoni* Toledano), or run on clay cliffs along roadsides (e.g., *Bembidion agonoides* Vigna Taglianti and Toledano, *Bembidion chimborazonum* Bates, *Bembidion walterrossii* Toledano), or inhabit open high-elevation grasslands (e.g., *Bembidion humboldti* Moret and Toledano, *Bembidion guamani* Moret and Toledano, *Bembidion chimborazonum* Bates, *Bembidion cotopaxi* Moret and Toledano).

In Toledano’s (2008) study on the Northern Andean fauna of *Bembidion*, he illustrated (his Figure 21) a single female belonging to the *georgeballi* species group from Rio Chalpi, Ecuador, noting that it was similar to *Bembidion georgeballi* but that it may represent a separate species; the decision about its status was postponed until males could be discovered. In 2010, the senior author and colleagues collected specimens of this form from the same region from which Toledano’s specimen originated. The larger series and characteristics of the male genitalia indicate that this form is a species distinct from *Bembidion georgeballi*, and it is here described, and compared to other members of the *georgeballi* group.

## Methods

Several hundred specimens of *Ecuadion* were examined as part of this study, including 50 specimens of *Bembidion georgeballi* and 16 specimens of *Bembidion ricei*, sp. n. Specimens came from or have been deposited in the collections listed below. Each collection’s listing begins with the coden used in the text.

**BMNH** The Natural History Museum, London

**CTVR** Luca Toledano Collection, Verona, Italy

**MNHN** Muséum National d’Histoire Naturelle, Paris

**OSAC** Oregon State Arthropod Collection, Oregon State University, Corvallis

**QCAZ** Catholic University of Ecuador, Quito

**USNM** National Museum of Natural History, Smithsonian Institution, Washington

Methods of specimen preparation for morphological work, and terms used, are given in Maddison (1993; 2008). Measurements for Apparent Body Length (ABL) are from apex of the labrum to apex of the longer elytron.

Photographs of body parts were taken with a Leica Z6 and JVC KY-F75U camera. For pronotal, elytral, and genitalic images, a stack of photographs at different focal planes was taken using Microvision’s Cartograph software; these photographs were then merged using the PMax procedure in Zerene Systems’s Zerene Stacker; the images thus potentially have some artifacts caused by the merging algorithm.

Sequences of 28S ribosomal DNA and cytochrome oxidase I genes were obtained using the protocols given in Maddison (2012), and deposited in GenBank with accession numbers JX971116 and JX971117.

### Common morphological features and composition of the *georgeballi* species group

**Figure 1.**
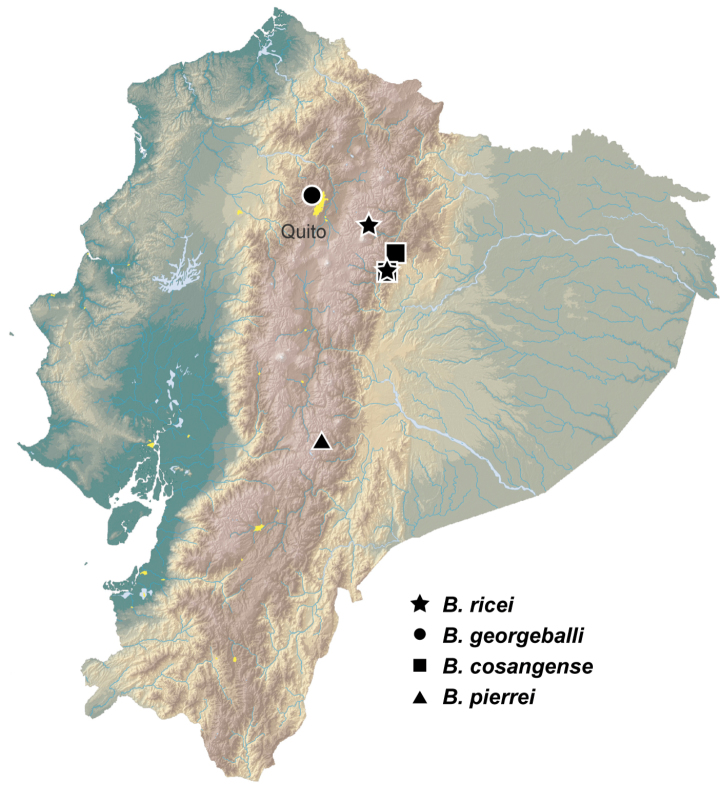
Geographic distribution of known members of the *georgeballi* species group in Ecuador. Base map modified from Chirico and Warner (2005).

Among *Ecuadion*, adults of the *georgeballi* group are characterized by convex elytral intervals, with deep and complete elytral striae, and with elytra reddish or with yellowish markings (Toledano 2008). There are five known species in the group, four of which are restricted to Ecuador (Fig. 1), the fifth in Venezuela:

*Bembidion georgeballi* Toledano [Ecuador]

*Bembidion ricei* Maddison and Toledano [Ecuador]

*Bembidion pierrei* Toledano [Ecuador]

*Bembidion cosangaense* Toledano [Ecuador]

*Bembidion guaramacal* Toledano [Venezuela]

### Species identification and description

**Table:** 

1	Elytra reddish, unicolorous; if with a faint, slightly darker spot, then with a pale rufous pronotum (see Toledano 2008: Fig. 24)	2
–	Elytra with a mottled testaceous and brown pattern, pronotum darker, at least centrally (Fig. 2)	3
2	Pronotum piceous-black, narrower (pronotal width/length = 1.17 to 1.20), elytra reddish, unicolorous; Ecuador	*Bembidion cosangaense*
–	Pronotum rufous, wider (pronotal width/length = 1.32 to 1.33), elytra reddish, sometimes with a faint, slightly darker spot; Venezuela	*Bembidion guaramacal*
3	Posterior lateral seta and carina of pronotum both absent, pronotum more constricted at hind angles (Fig. 3B)	*Bembidion georgeballi*
–	Posterior lateral seta and carina of pronotum both present, pronotum less constricted at hind angles (e.g., Fig. 3A)	4
4	Microsculpture absent from elytra in males and females; elytral striae 3 and 4 connected in front of the anterior discal seta (see Fig. 2A and 4A); elytral intervals notably convex	*Bembidion ricei* sp. n.
–	Isodiametric microsculpture on the whole dorsal surface in males and females; elytral striae normal; elytral intervals only slightly convex	*Bembidion pierrei*

#### Bembidion ricei

Maddison & Toledano sp. n.

urn:lsid:zoobank.org:act:A72AE0D9-3325-427C-B59B-E0B02507CA9B

http://species-id.net/wiki/Bembidion_ricei

##### Holotype.

Adult male, with three labels: “ECUADOR: Napo: Rio Chalpi Grande, 2800m, 0.3645°S, 78.0852°W, 26.x.2010. DRM 10.159. W.P. & D.R. Maddison, M. Reyes”, “David R. Maddison DNA2653 DNA Voucher [printed on pale green paper]”, and “HOLOTYPE *Bembidion ricei* Maddison & Toledano [printed on red paper]”. Specimen to be deposited at QCAZ; temporarily in OSAC. Genitalia in glycerine in vial pinned beneath specimen. GenBank accession numbers of DNA sequences from the holotype: JX971116 (28S ribosomal DNA) and JX971117 (cytochrome oxidase I).

##### Paratypes.

Six males and nine females from: ECUADOR: Napo: Rio Chalpi Grande, 2800m, 0.3645°S, 78.0852°W, 26.x.2010 & 8.xi.2010 (11 exx., OSAC, BMNH, MNHN); ECUADOR: Napo: Papallacta, 2750 m, Rio Chalpi, 8.xi.1985 (1 ex., CTVR); ECUADOR: Napo: Rio Guango (=Rio Huango), 2730m, 0.3758°S, 78.0748°W, 26.x.2010 (2 exx., OSAC); ECUADOR: Napo: Sierrazul (Hacienda Aragon) 10 km W of Cosanga, 2250m, 3–4.vi.1993, 00°44'08"S, 077°53'50"W (1 ex., USNM).

##### Type locality.

ECUADOR: Napo: Rio Chalpi Grande, 2800m, 0.3645°S, 78.0852°W.

##### Derivation of specific epithet.

It gives us great pleasure to name this species after the late Harold Edward Rice, a passionate butterfly collector and active member of the Pacific Northwest lepidopterist community, and friend to the senior author. Through Harold’s generosity, systematic entomology is well supported at Oregon State University. The fund he established paid for the expedition that yielded most of the known specimens of *Bembidion ricei*, including the holotype.

##### Diagnosis.

**Figure 2.**
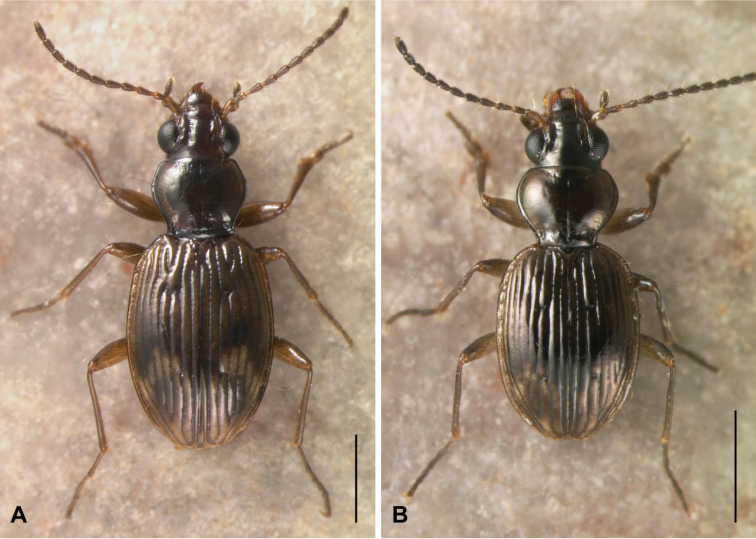
Habitus of male *Bembidion ricei* and *Bembidion georgeballi*. Scale bar is 1 mm. **A** *Bembidion ricei* (ECUADOR: Napo: Rio Chalpi Grande, 2800m, 0.3645°S, 78.0852°W, D.R. Maddison voucher V100622) **B** *Bembidion georgeballi* (ECUADOR: Pichincha: Quebrada Lozada, on road to Res. Yanacocha, 3460m, 0.1105°S, 78.5642°W, voucher V100658).

A shiny, medium-sized *Bembidion (Ecuadion)* with convex elytral intervals, and with a mottled pattern of light and dark on the elytra (as in Fig. 2A); adults have a seta at the hind corner of the pronotum, and lack elytral microsculpture in both males and females. Stria 3 and stria 4 are joined together and interrupted in front of the anterior discal seta (ed3; Fig. 2A, 4A); the striae are otherwise complete, and deeply engraved. This combination of characteristics is distinct within the genus.

**Figure 3.**
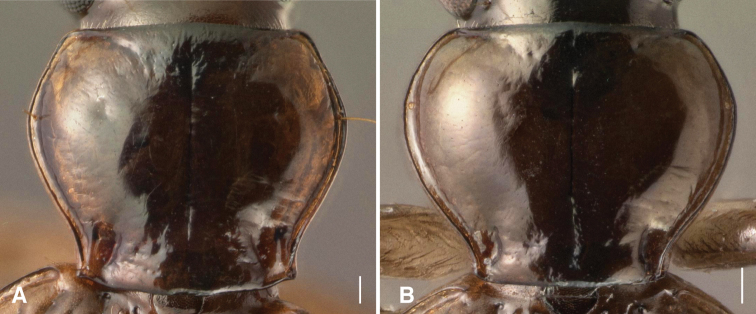
Pronota of male *Bembidion ricei* and *Bembidion georgeballi*. Scale bar is 0.1 mm. **A** *Bembidion ricei* (ECUADOR: Napo: Rio Chalpi Grande, 2800m, 0.3645°S, 78.0852°W, D.R. Maddison voucher V100677) **B** *Bembidion georgeballi* (ECUADOR: Pichincha: Quebrada Lozada, on road to Res. Yanacocha, 3460m, 0.1105°S, 78.5642°W, voucher V100658).

**Figure 4.**
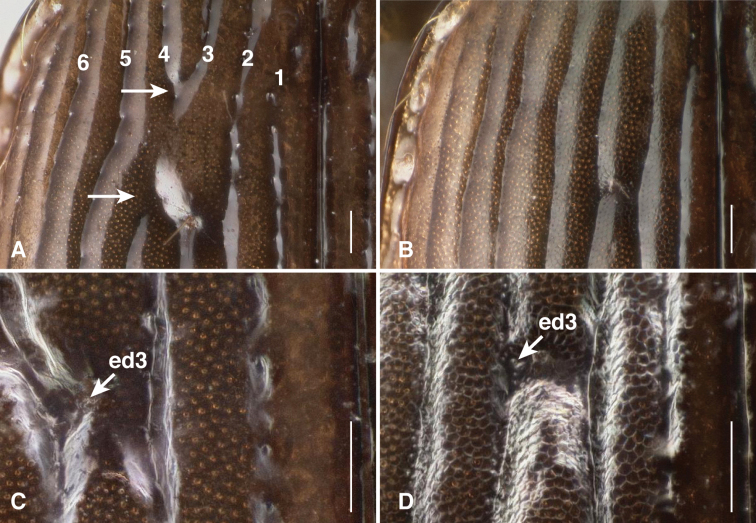
Elytra of *Bembidion ricei* (A & C, from ECUADOR: Napo: Rio Chalpi Grande, 2800m, 0.3645°S, 78.0852°W) and *Bembidion georgeballi* (B & D, from ECUADOR: Pichincha: Quebrada Lozada, on road to Res. Yanacocha, 3460m, 0.1105°S, 78.5642°W). Scale bar is 0.1 mm. **A** *Bembidion ricei* male (D.R. Maddison voucher V100656), showing joining of striae 3 and 4 (indicated by arrows), with subsequent gap in each stria (between arrows); illuminated by two diffuse lateral lights **B** *Bembidion georgeballi* female (voucher V100676); illuminated by two diffuse lateral lights **C** *Bembidion ricei* female (voucher V100675), illuminated by a ring light **D** *Bembidion georgeballi* female (voucher V100676), illuminated by a ring light.

**Figure 5.**
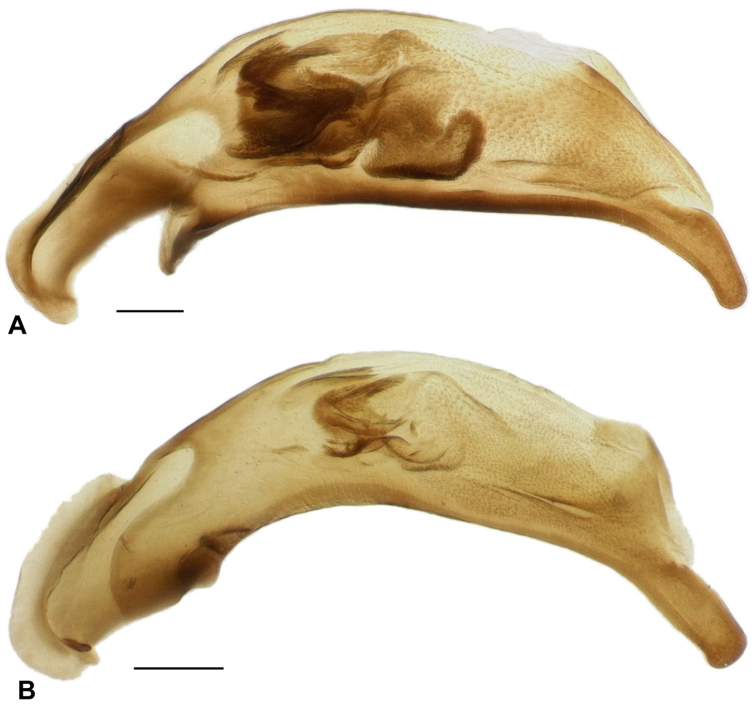
Male aedeagus. Scale bar is 0.1 mm. **A** *Bembidion ricei* (ECUADOR: Napo: Rio Chalpi Grande, 2800m, 0.3645°S, 78.0852°W, D.R. Maddison voucher V100656) **B** *Bembidion georgeballi* (ECUADOR: Pichincha: Campamento Pichán, 3350m, 0.1093°S, 78.5728°W, D.R. Maddison voucher V100657).

Brown, with lateral margins of pronotum paler brown in most specimens, and with elytra having a pale apex, and a pale transverse preapical region surrounded by darker brown (Fig. 2A); the region adjacent to ed3 is also slightly darker. Prothorax with sinuate lateral margin, hind angles about 90°, and with a posterior lateral carina (Fig. 3A). Elytral striae deep, complete, although with striae 3 and 4 joined together and interrupted in front of ed3 (Fig. 4A) in all 16 specimens examined; elytral intervals convex. Microsculpture absent from the pronotum and elytra in both sexes. Aedeagus as in Fig. 5A. ABL 3.9–4.1mm, n=6.

One of the more unusual aspects of these *Bembidion*, shared with some other *Ecuadion*, including males of *Bembidion georgeballi*, is the amber-like clarity of the elytra. The elytra are similar to clear lacquer in places, allowing bright microsculpture dots from the *undersurface* of the elytra to be visible dorsally (see pale dots in Fig. 4C).

The only other *Ecuadion* with yellow and brown mottled elytra, and with complete striae and convex elytral intervals, are *Bembidion georgeballi* and *Bembidion pierrei* (Toledano 2008). *Bembidion ricei* is most similar to *Bembidion georgeballi*, sharing more convex elytral intervals, and pale lateral regions of the pronotum in most specimens. Specimens differ in being larger (ABL 3.9–4.1mm in *Bembidion ricei*, 2.9–3.4mm in *Bembidion georgeballi*), with a less constricted posterior margin of the pronotum (compare Fig. 3A to 3B). The posterior lateral seta and carina of the pronotum are present (absent in *Bembidion georgeballi*). *Bembidion ricei* specimens have striae 3 and 4 joined and interrupted in front of discal seta ed3 (Fig. 4A). Microsculpture is lacking from the dorsal surface of the elytra in both males and females of *Bembidion ricei*, and thus they are very shiny (Fig. 4A, C); in *Bembidion georgeballi*, males lack elytral microsculpture, but females have evident isodiametric microsculpture throughout the elytra (Fig. 4B, D). In addition, the male aedeagus of *Bembidion ricei* has larger and darker sclerotized regions on the internal sac (Fig. 5A). *Bembidion georgeballi* is currently known only from 3350–3550m on the slopes of Volcán Guagua Pichincha west of Quito (Fig. 1).

From *Bembidion pierrei*, *Bembidion ricei* can be distinguished by having striae 3 and 4 joined, and lacking microsculpture on the elytra (*Bembidion pierrei* has isodiametric microsculpture throughout the elytra in both males and females). *Bembidion pierrei* also lacks the transparent, lacquer-like elytral regions of *Bembidion ricei*. *Bembidion pierrei* is known from the province of Chimborazo, far south of the localities of known localities of *Bembidion ricei* (Fig. 1).

##### Geographic distribution.

*Bembidion ricei* occurs in the province of Napo between 2250m and 2800m in the Andes of Ecuador, east of Quito (Fig. 1). Most specimens have been found a few kilometers east of Papallacta along two tributaries of the Rio Papallacta; a single female has been found along a tributary of the Rio Jondachi south of Cosanga and west of La Merced de Jondachi.

##### Habitat.

**Figure 6.**
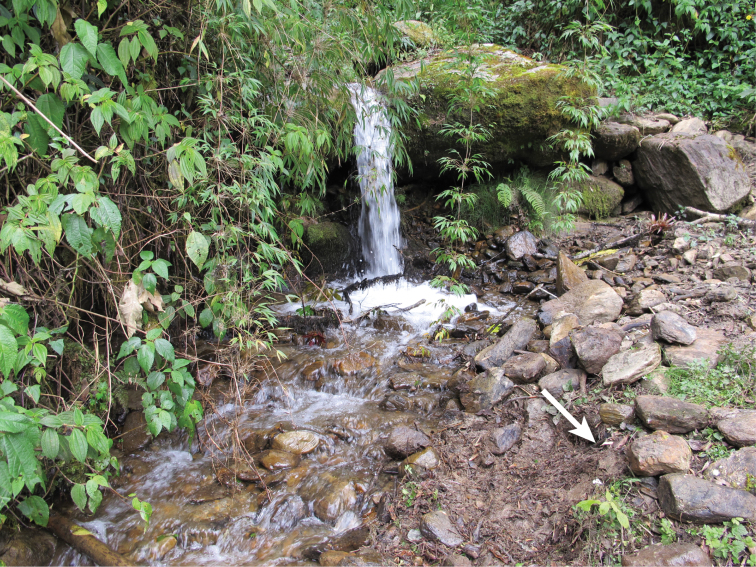
Type locality of *Bembidion ricei*, at Ecuador: Napo: Rio Chalpi Grande, 2800m, 0.3645°S, 78.0852°W. Specimens were found in damp leaf litter under rocks that had previously been at the site marked by the arrow; this is a small tributary of the Rio Chalpi Grande, within 4m of that river. Several *Andinodontis muellermotzfeldi* Toledano and Erwin were found within a meter of the *Bembidion ricei* habitat; *Bembidion (Ecuadion) sanctaemarthae* Darlington was common about 2–4m away, along the upper banks of the main river. One of the other known localities was along the same small creek, but upstream, in a more shaded area, and further away from the shore (about 1–2m from the water), among damp leaf litter and rocks.

Found among leaf litter and under rocks in moist areas near small streams in montane forest (Fig. 6). Specimens were found during daytime in leaf litter under rocks or by scratching open leaf litter.

## Supplementary Material

XML Treatment for Bembidion ricei
